# Supplementary material for: Wide Next-Generation Sequencing Characterization of Young Adults Non-Small-Cell Lung Cancer Patients
Source: Cancers (Basel). 2022 May 10;14(10):2352. doi: 10.3390/cancers14102352 (PMC9139648; doi:10.3390/cancers14102352)
Supplement: Supplementary file 1 [file cancers-14-02352-s001.zip › cancers-1632145-supplementary.pdf]

cancers

MDPI

Article

Wide Next-Generation Sequencing Characterization of Young Adults Non-Small-Cell Lung Cancer Patients

Paola Ulivi, Milena Urbini, Elisabetta Petracci, Matteo Canale, Alessandra Dubini, Daniela Bartolini, Daniele Calistri, Paola Cravero, Eugenio Fonzi, Giovanni Martinelli, Ilaria Priano, Kalliopi Andrikou, Giuseppe Bronte, Lucio Crinò and Angelo Delmonte

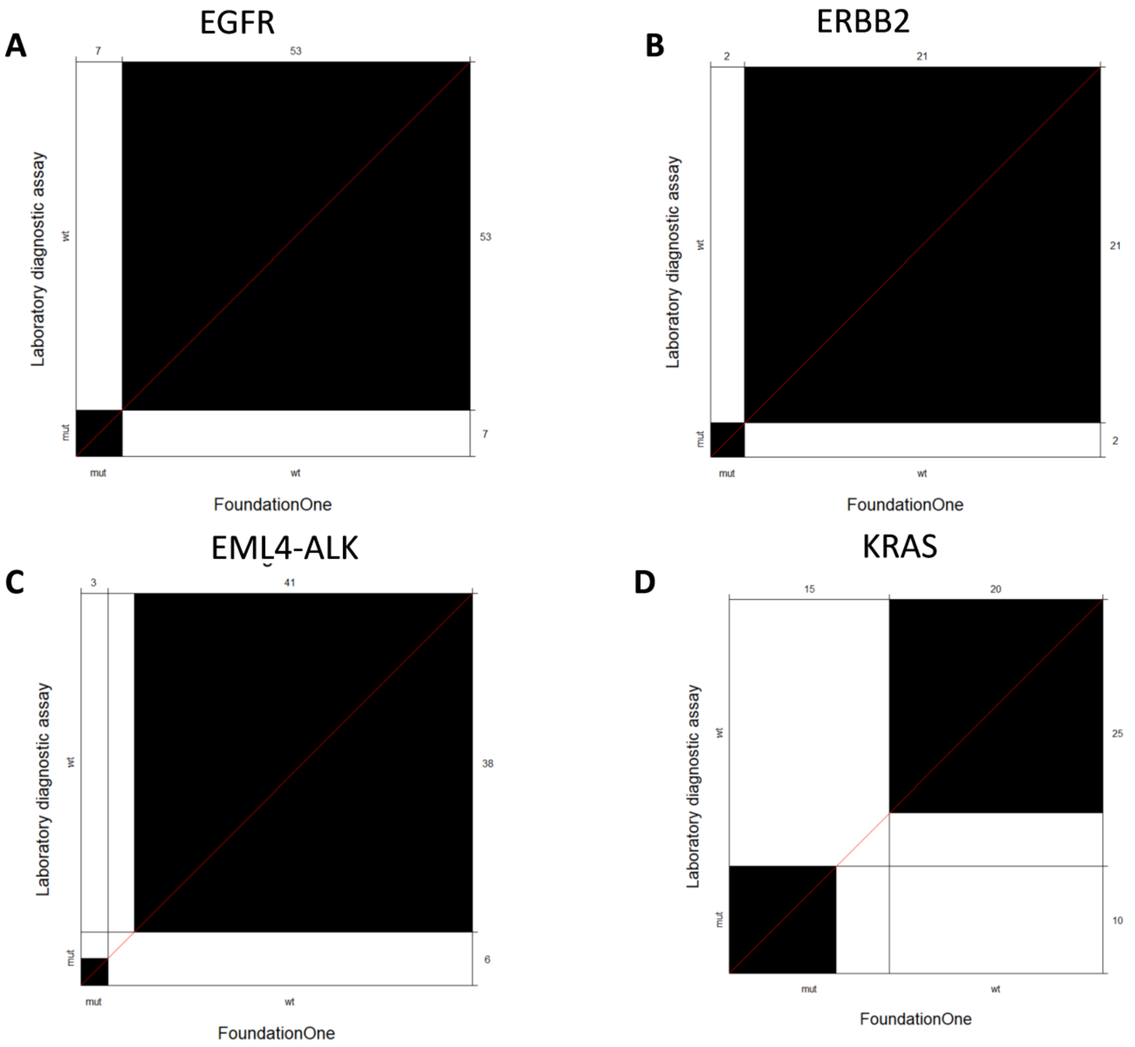

Figure S1. Agreement charts between Laboratory diagnostics test and FoundationOne assay for the main alterations: EGFR, KRAS, and HER2 mutations, and EML4-ALK translocations.

Table S1. Targettable gene variants.

Gene	N patients With Mutation	Exon Involved	Type of Mutation
EGFR	11	18	2 point mutations
		19	7 exon 19 deletions (3 with concomitant T790M)
		21	2 L858R
KRAS	36	2	15 G12C<br>5 G12D<br>5 G12V<br>2 G12A<br>1 G12F<br>1 G12S
		2	4 G13C<br>1 G13D
		3	2 Q61H
HER2	4	20	2 insertions<br>1 L755P
		19	1 S310F
BRAF	5	11	1 N581I<br>1 G469V<br>1G466E<br>D594N<br>G469A
PIK3CA	6	1	1 K111E
		9	3 E542K
		21	1 L1067F<br>1 H1047R<br>1 M1004I
MET	1	14	splice site 3028+1G>A
